# Genotyping-by-sequencing application on diploid rose and a resulting high-density SNP-based consensus map

**DOI:** 10.1038/s41438-018-0021-6

**Published:** 2018-04-01

**Authors:** Muqing Yan, David H. Byrne, Patricia E. Klein, Jizhou Yang, Qianni Dong, Natalie Anderson

**Affiliations:** 1 Department of Horticultural Sciences, Texas A&M University, College Station, TX 77843 USA; 2 Institute for Plant Genomics and Biotechnology, Texas A&M University, College Station, TX 77843 USA; 3 Present Address: Department of Computer Science, San Francisco State University, San Francisco, CA 94132 USA; 4 Present Address: Monsanto Company, 700 Chesterfield Parkway West, Chesterfield, MO 63017 USA

## Abstract

Roses, which have been cultivated for at least 5000 years, are one of the most important ornamental crops in the world. Because of the interspecific nature and high heterozygosity in commercial roses, the genetic resources available for rose are limited. To effectively identify markers associated with QTL controlling important traits, such as disease resistance, abundant markers along the genome and careful phenotyping are required. Utilizing genotyping by sequencing technology and the strawberry genome (*Fragaria vesca* v2.0.a1) as a reference, we generated thousands of informative single nucleotide polymorphism (SNP) markers. These SNPs along with known bridge simple sequence repeat (SSR) markers allowed us to create the first high-density integrated consensus map for diploid roses. Individual maps were first created for populations J06-20-14-3×“Little Chief” (J14-3×LC), J06-20-14-3×“Vineyard Song” (J14-3×VS) and “Old Blush”×“Red Fairy” (OB×RF) and these maps were linked with 824 SNPs and 13 SSR bridge markers. The anchor SSR markers were used to determine the numbering of the rose linkage groups. The diploid consensus map has seven linkage groups (LGs), a total length of 892.2 cM, and an average distance of 0.25 cM between 3527 markers. By combining three individual populations, the marker density and the reliability of the marker order in the consensus map was improved over a single population map. Extensive synteny between the strawberry and diploid rose genomes was observed. This consensus map will serve as the tool for the discovery of marker–trait associations in rose breeding using pedigree-based analysis. The high level of conservation observed between the strawberry and rose genomes will help further comparative studies within the Rosaceae family and may aid in the identification of candidate genes within QTL regions.

## Genetics: A DNA map of roses

A genetic map of rose DNA could help flower breeders develop ornamental crops that are more resistant to disease or have other desirable traits. Rose breeder, David Byrne and molecular geneticist, Patricia Klein along with their colleagues from Texas A&M University in College Station, USA, bred five different strains of ‘diploid’ rose, each with two full sets of chromosomes, to create 234 offspring plants. Using the full genome from strawberry, a closely related species, as a reference, the researchers then looked for sites in the genome where either single DNA letters differed between individual offspring or where short sequences of DNA repeated themselves to create easily identifiable genetic markers. They created genetic maps of each parental cross, and then formed a consensus map that can now serve as a tool for future genetically guided breeding efforts of horticulturally important traits.

## Introduction

Roses (*Rosa* spp.) are one of the most important and popular ornamental crops in the world today. Diverse plant growth types, flower colors, flower sizes/shapes, and fragrance all contribute to the commercial value of rose. Besides ornamental uses, roses also have medicinal, culinary, and cosmetic uses^1,2^. Rose is a very important ornamental plant in the US specialty crop market with an annual value of about $400 million^3^. There are ~200 *Rosa* species within the Rosaceae family of which about half are diploid (2*x* = 14). Among the more than 20,000 commercial rose cultivars^1^, most are either tetraploid (4*x* = 28), triploid (3*x* = 21), or diploid (2*x* = 14)^1,4^. Most cultivated roses are hybrids derived from 8 to 10 wild diploid and tetraploid species^5,6^. Though DNA amounts were found varying among diploid rose sections, subgenera and cultivars, the diploid rose genome size was reported to be small among the angiosperms, about 0.78–1.29 pg/2C, which is about two to four times the size of *Arabidopsis thaliana* (L.) Heynh^7–10^.

Genetic maps have been constructed in rose using a range of markers including phenotypic (i.e. visible) traits, isozymes, random amplified polymorphic DNAs (RAPDs), restriction fragment length polymorphisms (RFLPs), amplified fragment length polymorphisms (AFLPs), sequence-tagged sites (STSs), microsatellites or simple sequence repeats (SSRs), and single nucleotide polymorphisms (SNPs)^11–15^. Effective linkage map construction requires polymorphic markers, which are evenly distributed across the genome or the region of interest, high marker coverage, and a low genotyping error rate^16^. Initial linkage maps created for diploid roses started with creating two parental maps using the pseudo-testcross strategy, one for the female and the other for the male. In addition, these maps were created using relatively small populations (100 or less) due to the varying fertility and germination abilities of different rose genotypes. The first several diploid rose genetic maps utilized morphological markers as well as molecular markers and had from less than a hundred to about three hundred markers covering about 300–500 cM for each parental map^11,17–19^. Genetic map construction has also been conducted in tetraploid roses with various marker types^14,15,20^. More recently, the integrated map approach has been possible utilizing a greater number of markers resulting in longer map lengths. Linde et al.^21^ developed an integrated diploid genetic map for rose using 233 markers covering 418 cM of the rose genome. For tetraploid rose, Yu et al.^22^ integrated the homologous linkage groups from both parents with 74 SSRs and constructed an integrated map with length of 874 cM. Most recently, Vukosavljev et al.^14^ and Bourke et al.^15^ both created integrated linkage maps using the WagRhSNP 68K Axiom SNP array^23^. Beyond the individual maps, an unified diploid consensus map for rose was constructed in 2011 using 59 bridge markers to link four diploid rose populations^24^. This ICM (integrated consensus map) included 597 markers and covered a length of 530 cM on seven linkage groups. These mapping studies also revealed genes or QTLs associated with horticultural traits such as thorn density, leaf area, chlorophyll content, flower size, days to flowering, leaf size, and resistance to powdery mildew^19,21,25^.

Genomic comparative studies within the Rosaceae family have shown that the synteny and collinearity among *Prunus*, *Malus*, *Pyrus*, *Fragaria*, and *Rosa* is high^14,15,20,26–30^. Strawberry and rose both belong to the Rosoideae subfamily of the Rosaceae with a base chromosome number of 7, and they have been shown to have a close genetic relationship^14,15,20,24,31^. Gar et al.^20^ compared the collinearity among *Rosa* and *Fragaria* by positioning 70 rose EST markers on the strawberry pseudo-chromosomes. They found most of the markers mapped to one linkage group of *Rosa* were located on one *Fragaria* pseudo-chromosome. It was estimated that four major translocations and six inversions have occurred between the *Rosa* and *Fragaria* genomes since their divergence from a common ancestor about 62–82 million years ago^32^. With the recent release of a new version of the diploid *Fragaria vesca* genome v2.0.a1 (denoted as Fvb)^33–35^ and improved sequencing technologies, synteny between *Rosa* and *Fragaria* can now be examined at a much higher resolution. In recent studies, the comprehensive collinearity between strawberry and rose was demonstrated utilizing the WagRhSNP 68K Axiom SNP array on tetraploid roses^14,15^. These studies described the detailed syntenic relationship between the seven strawberry and rose chromosomes, and revealed a reciprocal translocation, a major telomeric inversion, and another possible inversion differentiating the chromosomes of these two genera^14,15^.

Currently, it is possible to sequence whole-plant genomes or sample the genome (or transcriptomes) more thoroughly and cost-efficiently using next-generation sequencing (NGS) technologies such as Roche 454, Illumina, AB SOLiD, and PacBio RS^36^. Although biallelic SNP markers generated from genotyping by sequencing (GBS) contain less information than multi-allelic markers and a relatively high error rate^16^, their abundance and cost effectiveness^37,38^ have facilitated the genomic study of many plant species, including Thale cress (*Arabidopsis thaliana* L.)^39^, maize (*Zea mays* L.)^40^, rice (*Oryza sativa* L.)^41^, sorghum (*Sorghum bicolor* L.*)*^42^, and barley (*Hordeum vulgare* L.)^43^, as well as many heterozygous horticultural crops such as apple (*Malus*×*domestica* Borhk)^44^, grapevine (*Vitis vinifera* L.)^45^, strawberry (*Fragaria iinumae* Makino)^46^, sweet cherry (*Prunus avium* L.)^26^, and tetraploid cut roses (*Rosa hybrid* L.)^13^.

The aim of this study was to use previously developed anchor SSRs^24^ and SNPs generated from GBS to construct a dense integrated consensus map for several diploid rose populations. This diploid rose consensus map (ICD) enabled us to visualize the syntenic relationship between strawberry and diploid rose, and compare and validate marker order across populations. The development of a high-density consensus genetic map in rose will help identify QTLs, candidate genes, benefit marker-assisted selection, and facilitate the study of syntenic relationships across taxonomic groups.

## Materials and methods

### Mapping populations

A highly black spot resistant breeding line derived from *R. wichurana* “Basye’s Thornless” (black spot resistant)—J06-20-14-3 (J14-3) according to Dong et al.^47^, a moderately resistant cultivar “Old Blush” (OB) and three susceptible cultivars with excellent ornamental characteristics—“Little Chief” (LC), “Red Fairy” (RF), and “Vineyard Song” (VS) were used to generate the three diploid populations (2*n* = 2*x* = 14) for linkage map construction (Table 1). Parents J14-3, OB, LC, RF, and VS also diverge in growth habit, horticultural characteristics and heat tolerance. These populations were grown in the field in College Station (30°36′5″N 96°18′52″W, 112 m elevation), TX, USA, a subtropical mild winter, hot summer humid climate, which has an average annual rainfall of 1018 mm, and Spring, Summer, Fall, and Winter average temperatures of 20, 29, 21, and 12 °C, respectively^48^. One plant per seedling was planted on raised beds in rows oriented east to west in an open field in 2013 or 2014. Black landscape cloth weed barrier was placed around each plant for weed control. Each plant was hard pruned (reduced plant size by 50–75%) at the end of the winter in February/March and light pruned (reduced plant size by 25–40%) in both June and September to restrict plant size and induce new growth. Irrigation was applied as needed, but no chemical applications were applied.

**Table 1:** Diploid rose parents of the three mapping populations and their response to black spot disease

Female parent	Male parent	Population size
J06-20-14-3 (HR)	“Little Chief” (S)	69
J06-20-14-3 (HR)	“Vineyard Song” (S)	83
“Old Blush” (MR)	“Red Fairy” (S)	82

*S* susceptible, *MR* moderate resistance, *HR* high resistance

### DNA extraction

DNA extraction was performed based on Doyle’s^49^ CTAB protocol with some minor modifications. The stock solution preparation and DNA extraction protocol can be found in Supplementary File 1. Unexpanded young leaves were collected up to 1/3 volume of a 2 mL screw-cap tube and placed in liquid nitrogen immediately and stored at −80 °C until extraction. After extraction, DNA samples were incubated with RNase at 37 °C for forty to 50 min and then the isolated genomic DNA was purified using the OneStep™ PCR Inhibitor Removal Kit (Zymo Research, Irvine, CA, USA) according to the manufacturer’s protocol. DNA quantification was performed fluorometrically using a Qubit Fluorometer (Thermo Fisher Scientific, Rochester, NY, USA) or AccuBlue™ (Biotium, Hayward, CA, USA) according to the protocol from the manufacturer. All DNA samples were stored at −20 °C.

### SSR analysis

Forty SSR markers described by Spiller et al.^24^ as bridge markers were analyzed on the five parental lines: J14-3, OB, RF, VS, and LC. The original SSR names were appended to include the ICM LG numbers^24^. Twenty-six (Table 2) of the 40 SSRs were polymorphic within the three mapping populations and were run on the progenies to determine the linkage groups according to the rose ICM^24^ and used as quality control markers. The 10 µL PCR reaction mixture contained 2 µL of 2.5 ng/µL genomic DNA, 2 µL 5×GoTaq Reaction Buffer (Promega Corporation, Madison, WI, USA), 1 µL 25 mM dNTP, 0.8 µL 25 mM MgCl_2_, 1 µL HEX, TET, FAM, or NED fluorescently labeled forward primer, 1 µL non-fluorescently labeled reverse primer, 0.04 µL GoTaq DNA polymerase (Promega Corporation), and 2.16 µL autoclaved DNase-/RNase-free water. The PCR reactions were performed in a GeneAmp® 9700 thermal cycler (Applied Biosystems, Foster City, CA, USA) using the following program: denaturation at 95 °C for 2 min, followed by 25 cycles of denaturation at 95 °C for 45 s, primer annealing at 55 °C for 45 s, and primer extension at 72 °C for 1 min. A final extension was carried out at 72 °C for 20 mins, and then held at 4 °C. PCR product (1 µL) was then added to 5 µL Hi-Di Formamide and ROX400 master mix (1 mL Hi-Di Formamide + 50 µL ROX400), followed by denaturation at 95 °C for 5 mins. The mixture was run on an ABI3130xl Genetic Analyzer (Applied Biosystems). The files generated by the ABI3130xl were then analyzed using GeneMapper v4.0 (Applied Biosystems). The allele sizes called from GeneMapper were converted into JoinMap® v4.1^50^ CP (cross pollination) standard codes: abxcd, hkxhk, lmxll, and nnxnp.

**Table 2:** Anchor SSR markers tested on the three diploid rose mapping populations

Mapping populations	Number of SSRs	Names of SSR			
J14-3×LC	15	CL2845_LG5	CL2980_LG6	*CL3881_LG4*	H5_F12_LG1
		Rh48_LG2	Rh72_LG7	RhAB9-2_LG1	RhABT12_LG4
		RMS043_LG7	Rw12J12_LG3	Rw14H21_LG5	Rw22B6_LG7
		Rw34L6_LG1	Rw35C24_LG3	Rw5G14_LG7	
J14-3×VS	17	CL2845_LG5	CL2980_LG6	*CL3881_LG4*	*CTG21_LG3*
		H5_F12_LG1	Rh48_LG2	*Rh58_LG3*	Rh72_LG7
		RhABT12_LG4	RMS001_LG7	Rw12J12_LG3	Rw14H21_LG5
		Rw22B6_LG7	Rw34L6_LG1	Rw35C24_LG3	*Rw55E12_LG4*
		Rw5G14_LG7			
OB×RF	21	BFACT47_LG3	CL2980_LG6	CL2996_LG2	*CTG21_LG3*
		H5_F12_LG1	Rh48_LG2	Rh50_LG3	Rh72_LG7
		*Rh93_LG5*	RhAB9-2_LG1	RhABT12_LG4	RMS003_LG7
		RMS015_LG1	RMS043_LG7	*Rw11E5_LG6*	Rw12J12_LG3
		Rw14H21_LG5	Rw22B6_LG7	Rw34L6_LG1	Rw35C24_LG3
		Rw5G14_LG7			

Italicized SSR markers were grouped into the expected LGs but not included in the final map

### Genotyping by sequencing and SNP detection

Genotyping by sequencing or digital genotyping was performed using the methylation sensitive restriction enzyme *Ngo*MIV (G^˅^CCGGC) according to the method described by Morishige et al.^42^ Briefly, 250 ng rose DNA was digested with the restriction enzyme *Ngo*MIV. Following digestion, multiplex identifier barcodes were ligated to the fragments, which were subsequently grouped into pools of 66 samples, each containing a unique 12 bp barcode. The pools were sheared by sonication to a target size of 250–300 bp followed by size selection on a 2% agarose gel. Following overhang fill-in, blunting and adenylation, the pools underwent ligation with an Illumina-specific adapter and were purified using Agencourt AMPure XP magnetic beads (Beckman Coulter, Indianapolis, IN, USA). The pools were then subjected to 20 cycles of PCR using Phusion high-fidelity polymerase (Thermo Fisher Scientific). Single-strand products were obtained using Dynabeads® (Thermo Fisher Scientific) then PCR-amplified for 14 cycles with Phusion polymerase to incorporate the Illumina bridge amplification sequence. Final PCR products were purified then quantified using PicoGreen® fluorescent dye (Quant-iT™ dsDNA Broad Range (BR) kit, Thermo Fisher Scientific). Final PCR products were diluted to 10 nM. Quality assessment of each template library was performed using an Agilent 2100 Bioanalyzer (Agilent Technologies, Santa Clara, CA, USA). The template was sequenced on an Illumina HiSeq 2500 (Illumina, San Diego, CA, USA) using standard Illumina protocols. Single-end sequencing was carried out for 126 cycles. Only Illumina data that passed quality control (FastQC) was further analyzed. Reads for each parent and progeny were identified by their unique 12 bp barcode identifier and sorted into individual files using a custom python script. A 100% match to both the 12 bp barcode sequence and the partial *Ngo*MIV restriction site were crucial to retain the reads from each sample. Following the sorting of reads to each individual sample, the 12 bp barcode on the 5′ end was trimmed and the reads were imported into the CLC Genomics Workbench v9.0 (Qiagen, Boston, MA, USA). Trimmed reads from each sample were mapped to the *Fragaria vesca* genome v2.0.a1 (Fvb)^34^. Parameters for read alignment were set at a mismatch cost = 2, insertion and deletion cost = 3, 50% minimum read length required to match the reference and a minimum of 75% similarity between the reads and the reference genome. Any reads that failed to align to the reference genome or aligned identically to more than one position were ignored. After the alignment, variant detection was performed to call SNPs. The parameters for SNP detection in the CLC Genomics Workbench were: at least 90% probability to detect a variant, a minimum read coverage of 15 to detect a SNP, a minimum SNP count of 3, a neighborhood radius = 5, a minimum central quality = 20, a minimum neighborhood quality = 15. These parameters were applied to determine legitimate SNPs. The mapping and SNP files were exported as SAM and comma-separated-value (.csv) formats, respectively. Further SNP call analysis was performed using custom scripts written in python and perl. The scripts used for the GBS pipeline can be found in the Dryad Digital Depository, doi:10.5061/dryad.k2do5. SNP markers were named according to their physical position on the *Fragaria vesca* whole-genome v2.0.a1 Assembly & Annotation in the GDR database^51^. For example, SNP chr1_19.680628 is located on *Fragaria vesca* pseudo-chromosome 1 at position 19.680628 Mbp. Marker alleles were converted into genotype codes based on the possible CP population segregation types abxcd, efxeg, hkxhk, lmxll, and nnxnp as described in the JoinMap® v4.1^50^ manual using a custom python script.

### Individual genetic linkage map construction

Individual linkage maps were first developed from the crosses of J14-3×LC, J14-3×VS, and OB×RF independently using JoinMap® v4.1. SNPs were eliminated if both parents were homozygous, if one or both parents had no allele call at a given position, if there was too much missing data (>15% of the population size), if the segregation ratio was heavily skewed based on a *χ*^2^ test (*p* ≤ 0.0005), or if any parental genotype did not follow what was described as a CP population as outlined in the JoinMap® v4.1 manual^50^. For the purpose of constructing the consensus rose map, after the application of the filtering criteria mentioned above and before importing markers into JoinMap® v4.1, 1014 SNPs that were common across all three diploid populations were appended with a “c” at the end of the marker name and the best effort to retain them throughout the mapping process was implemented regardless of the similarity of their segregation patterns. As for the rest of the markers, only one was kept if it co-segregated with other markers. Markers were grouped to the seven rose linkage groups with different LOD values that varied from 5 to 15 (9, 11, 5, 9, 11, 11, 11; 7, 7, 7, 11, 11, 7, 7; 15, 15, 14, 15, 14, 15, 14 for the seven LGs of OB×RF, J14-3×LC, and J14-3×VS, respectively). Each group was assigned to one of the seven rose linkage groups according to the anchor SSR markers with previously known linkage group positions^24^. The maps were constructed with the maximum likelihood mapping function. Poorly fitting markers that greatly inflated the map length or resulted in too many double recombinations were dropped during the mapping process. Graphical genotyping in Excel (E. van de Weg, personal communication (2016)) was used to check marker double recombinations. In addition, individuals with many unexpected alien alleles (>3%) or too many recombination events (either outcrosses or selfed progeny) were dropped before the final mapping. The common markers excluded during the mapping process were placed back onto the map in the final step after fixed-ordering all other markers to facilitate map integration across the three populations. The final linkage maps were visualized with MapChart 2.3^52^.

### Integrated consensus map construction and synteny comparison

After each map was constructed for a population, a total of 234 F_1_ progeny with 824 common SNPs and 13 common SSRs were used for developing an integrated consensus map. Map integration was first attempted using the JoinMap® v4.1 “combine groups for map integration” function, however, due to reshuffling of marker order within each individual map and extremely long computational time resulting from the large number of markers, consensus map construction within JoinMap® v4.1 was difficult. Therefore, MergeMap^53^ was used to generate consensus marker order using homologous LGs from individual maps. The consensus map created in MergeMap was of higher quality than the consensus map created in JoinMap® v4.1 (data not shown) with regard to marker number and marker density. The integrated consensus map for the three diploid populations was designated as ICD (integrated consensus map for diploid rose).

Genomic comparison between diploid rose and *F. vesca* was performed following construction of the rose ICD map. The comparison of these two genera was visualized using Circos^54^ diagrams.

## Results

### Mapping materials

Among all three mapping populations, 19 individuals were excluded during marker analysis and mapping due to an excessive number of alien alleles (suspicious outcrosses, >3%) or selfing events causing too many double recombinations. As a result, a total of 234 plants plus five parental lines were used to develop the linkage maps (Table 1).

### Anchor SSR markers

Twenty-six out of 40 tested anchor SSRs were polymorphic in all three populations and thus used as quality control markers (Table 2; Supplementary Table 1), among which, six SSR markers failed to fit in the final maps though they were initially grouped into the expected LGs along with the SNP markers. On the final individual maps, 14, 13, and 18 SSRs were incorporated into the J14-3×LC, J14-3×VS, and OB×RF maps, respectively (Table 2). These SSRs were distributed on all 7 LGs allowing us to assign each of the LGs according to the rose ICM^24^. After integrating all three maps, 20 SSR markers were present on the ICD, whereas 13 of them were present on at least two maps and 10 were shared across all three (Supplementary Figures 1–8).

### SNP markers

The parents and progeny from the 3 rose populations were run on 5 lanes of an Illumina flow cell. A total of ~99 Gb of sequence was obtained. Of the 255 progeny and 5 parents originally sequenced, only two progeny failed to sequence and these were removed from further analysis in addition to the 19 that were excluded during the mapping process as noted above. On average, 3.3 M reads were obtained for each sample and approximately 60.4% of the reads from each sample mapped to the *F. vesca* reference genome. After calling variants in the CLC Genomics Workbench, we initially obtained more than fifty thousand SNPs for each population (data not shown). However, after removing SNPs that were monomorphic, had too much missing data (>15% of population size), or the marker genotypes were not described in the JoinMap® v4.1 manual^50^, we retained ~7000 SNPs per population. An additional two thousand SNPs were eliminated due to strong segregation distortion (*p* < 0.0005) leaving ~5000 candidate SNPs, including 1014 that were common among the three populations, for mapping. During the mapping process, ~3500 SNPs were eliminated because of co-segregation or because they failed to fit in the final map. Fourteen to fifteen hundred SNPs were successfully mapped to each population with hundreds of SNPs placed on each LG (Table 3). Among these, 824 SNPs common in at least two populations (192 common in all three) were retained to aid in map integration. The allele calls for the markers retained for each of the three individual mapping populations can be found in Supplementary Table 2.

**Table 3:** SSR, SNP, distorted markers, and bin markers mapped to each LG for the three diploid rose population as well as the integrated consensus map

		Population			
LG	Marker statistics	J14-3×LC	J14-3×VS	OB×RF	ICD
LG1	SSR no.	3	2	4	4
	SNP no.	186	159	99	344
	Distorted marker (*p* < 0.05)	0	**60**	48	—
	Bin marker no.	38	39	48	93
	Total	189	161	103	348
LG2	SSR no.	1	1	2	2
	SNP no.	270	296	367	751
	Distorted marker (*p* < 0.05)	**81**	7	**53**	—
	Bin marker no.	60	81	76	161
	Total	271	297	369	753
LG3	SSR no.	2	2	4	4
	SNP no.	194	121	80	336
	Distorted marker (*p* < 0.05)	22	27	14	—
	Bin marker no.	49	43	31	91
	Total	196	123	84	340
LG4	SSR no.	1	1	1	1
	SNP no.	198	223	220	519
	Distorted marker (*p* < 0.05)	32	4	0	—
	Bin marker no.	40	49	61	120
	Total	199	224	221	520
LG5	SSR no.	2	2	1	2
	SNP no.	273	224	302	562
	Distorted marker (*p* < 0.05)	29	**60**	0	—
	Bin marker no.	50	56	64	121
	Total	275	226	303	564
LG6	SSR no.	1	1	1	1
	SNP no.	219	139	224	471
	Distorted marker (*p* < 0.05)	**52**	10	**87**	—
	Bin marker no.	45	39	61	109
	Total	220	140	225	472
LG7	SSR no.	4	4	5	6
	SNP no.	227	259	241	524
	Distorted marker (*p* < 0.05)	0	32	24	—
	Bin marker no.	54	62	45	125
	Total	231	263	246	530
Overall	SSR no.	14	13	18	20
	SNP no.	1567	1421	1533	3507
	Distorted marker (*p* < 0.05)	216	200	226	—
	Bin marker no.	336	369	386	820
	Total	1581	1434	1551	3527

Marker distortion was based on a *χ*^2^ test (*p* < 0.05); LGs having more than 50 highly distorted markers are shown in bold for each population; “—” indicates distortion is not available for the consensus map.

### Individual linkage map construction

A total of 14 SSR and 1567 SNP markers were mapped in the J14-3×LC population (464 cM) (Supplementary Figures 3 and 4), 13 SSR and 1421 SNP markers were mapped in the J14-3×VS population (518 cM) (Supplementary Figures 5 and 6), and 18 SSR and 1533 SNP markers were mapped in the OB×RF population (524 cM) (Supplementary Figures 7 and 8). Mean distance was calculated using the unique loci, where co-segregating markers were considered as one bin marker. The map density and mean distance across all the LGs varied from 1 to 4 markers per cM and 1–2.19 cM/bin marker, respectively. The largest gaps ranged from 3 to 15 cM (Table 4). Across the three populations, 837 markers (SNP + SSR) were shared between at least two populations and 203 markers (SNP + SSR) were shared across all three populations. These anchor markers were used to integrate the three individual maps.

**Table 4:** Statistical summary of the individual diploid rose maps and the integrated consensus map by linkage group (LG)

		Linkage groups							
Population	Map	LG1	LG2	LG3	LG4	LG5	LG6	LG7	Overall
J14-3×LC	Map length (cM)	50.5	74.8	62.1	53.0	74.3	76.8	72.5	464.0
	Map density (markers/cM)	3.7	3.6	3.2	3.8	3.7	2.9	3.2	3.4
	Mean distance (cM/bin marker)	1.3	1.3	1.3	1.3	1.5	1.7	1.3	1.4
	Largest gap (cM)	3.0	3.8	4.5	3.8	5.2	13.1	4.3	13.1
J14-3×VS	Map length (cM)	51.8	84.9	79.6	55.4	100.6	67.8	77.7	517.8
	Map density (markers/cM)	3.1	3.5	1.6	4.0	2.3	2.1	3.4	2.8
	Mean distance (cM/bin marker)	1.3	1.1	1.9	1.1	1.8	1.7	1.3	1.4
	Largest gap (cM)	3.8	5.7	8.5	4.2	12.3	5.1	4.4	12.3
OB×RF	Map length (cM)	65.5	83.6	67.8	61.0	80.3	90.2	75.7	524.1
	Map density (markers/cM)	1.6	4.4	1.2	3.6	3.8	2.5	3.3	3.0
	Mean distance (cM/bin marker)	1.4	1.1	2.2	1.0	1.3	1.5	1.7	1.4
	Largest gap (cM)	4.6	4.3	6.9	2.8	3.8	14.7	6.3	14.7
ICD	Map length (cM)	94.6	133.0	118.2	117.3	152.7	109.5	166.9	892.2
	Map density (markers/cM)	3.7	5.7	2.9	4.4	3.7	4.3	3.2	4.0
	Mean distance (cM/bin marker)	1.0	0.8	1.3	1.0	1.3	1.0	1.3	1.1
	Largest gap (cM)	3.8	4.5	8.5	4.2	11.2	3.6	5.9	11.2

Around 14% of the mapped markers showed segregation distortion (0.0005 < *p* < 0.05) with the distortion ratio varying among LGs and populations (Table 3). Segregation distortion was predominantly clustered into regions on LGs 2 and 6 of the J14-3×LC population, LGs 1 and 5 of the J14-3×VS population, and LGs 1, 2, and 6 of the OB×RF population (Table 3; Supplementary Figures 3–8). In total, 19 markers (3 SSR and 16 SNP) showed segregation distortion (*p* < 0.05) in two populations and none of the markers showed distortion in all three populations (data not shown). Overall, 206, 187, and 211 markers showed high skewness only in the J14-3×LC, J14-3×VS, and OB×RF populations, respectively (data not shown). The majority of the markers on the final individual population maps passed the goodness-of-fit test favoring the alleles from both parental lines, which indicates a good level of cross and self-compatibility among the parental materials.

### Integrated consensus map for diploid rose (ICD) construction

**Fig. 1:**
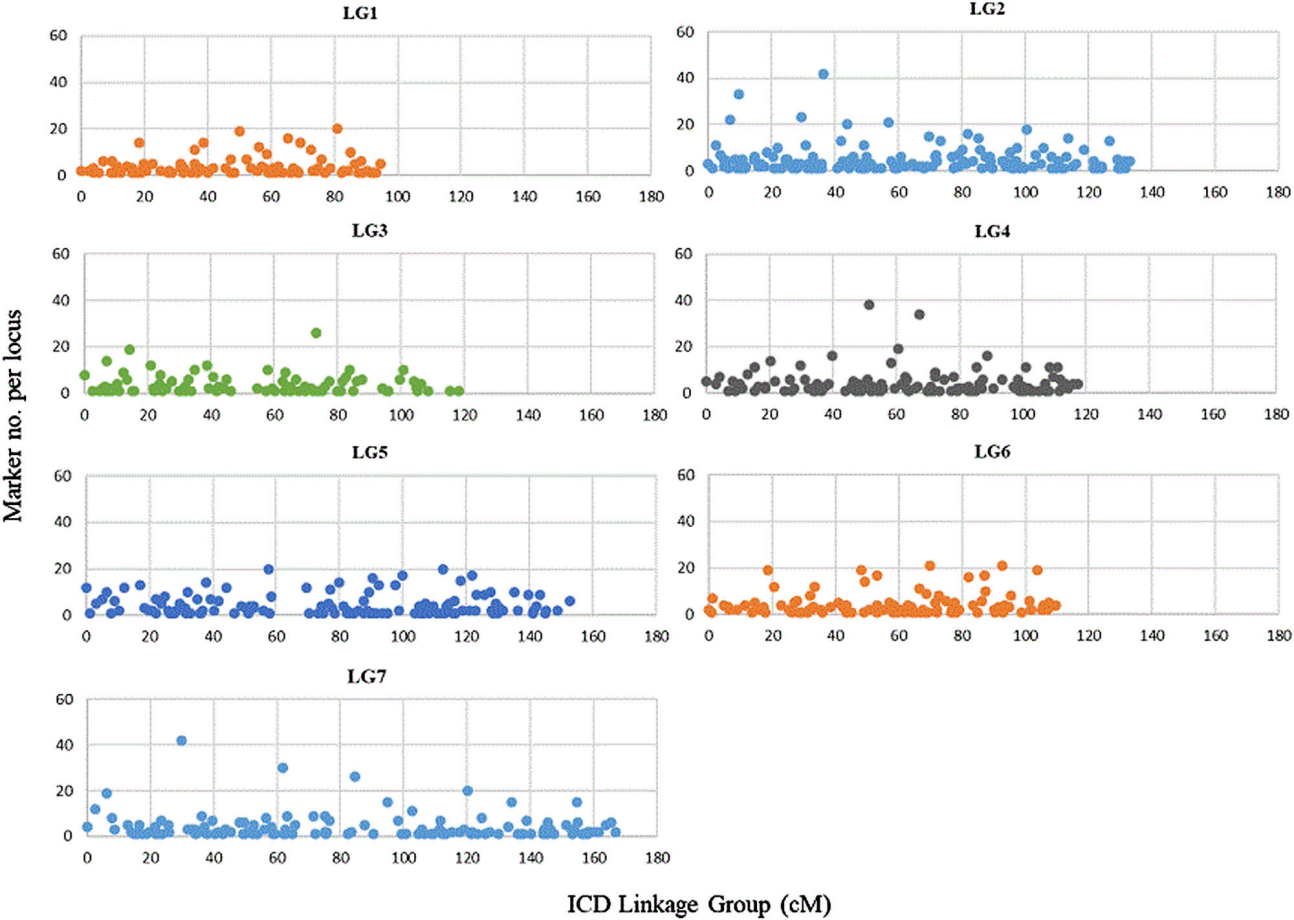
Marker density and distribution along the seven LGs of the diploid rose ICD. The length (cM) of each LG is shown along the *X* axis and the number of co-segregating markers per locus are shown on the *Y* axis

The ICD was developed by combining the marker data from the three individual populations. Thirteen SSR and 824 SNP markers shared between at least two populations served as bridge markers to integrate the individual maps resulting in a consensus map with 3527 markers (20 anchor SSRs and 3507 SNPs) and a map length of 892 cM (Tables 3 and 4; Supplementary Figures 1 and2). The largest gap in the ICD was 11.2 cM on LG5. Overall marker density was 4 markers/cM, and there was, on average, one bin marker every 1 cM. The LGs ranged in size from 95 to 167 cM and marker number varied from 300 to 700. The largest linkage group was LG7 (167 cM) but LG2 had the highest marker density (6 markers/cM) and the least mean distance (1 cM/bin marker) among bin markers (Table 3; Supplementary Figure 9). Compared to the individual maps, the total map length was increased by nearly 390 cM although the map density and mean distance between markers was improved. Many markers were mapped to the same locus due to their identical or similar segregation patterns and this occurred on every linkage group with as many as 40 markers co-segregating at one locus (LGs 2, 4, 7) (Fig. 1).

**Fig. 2:**
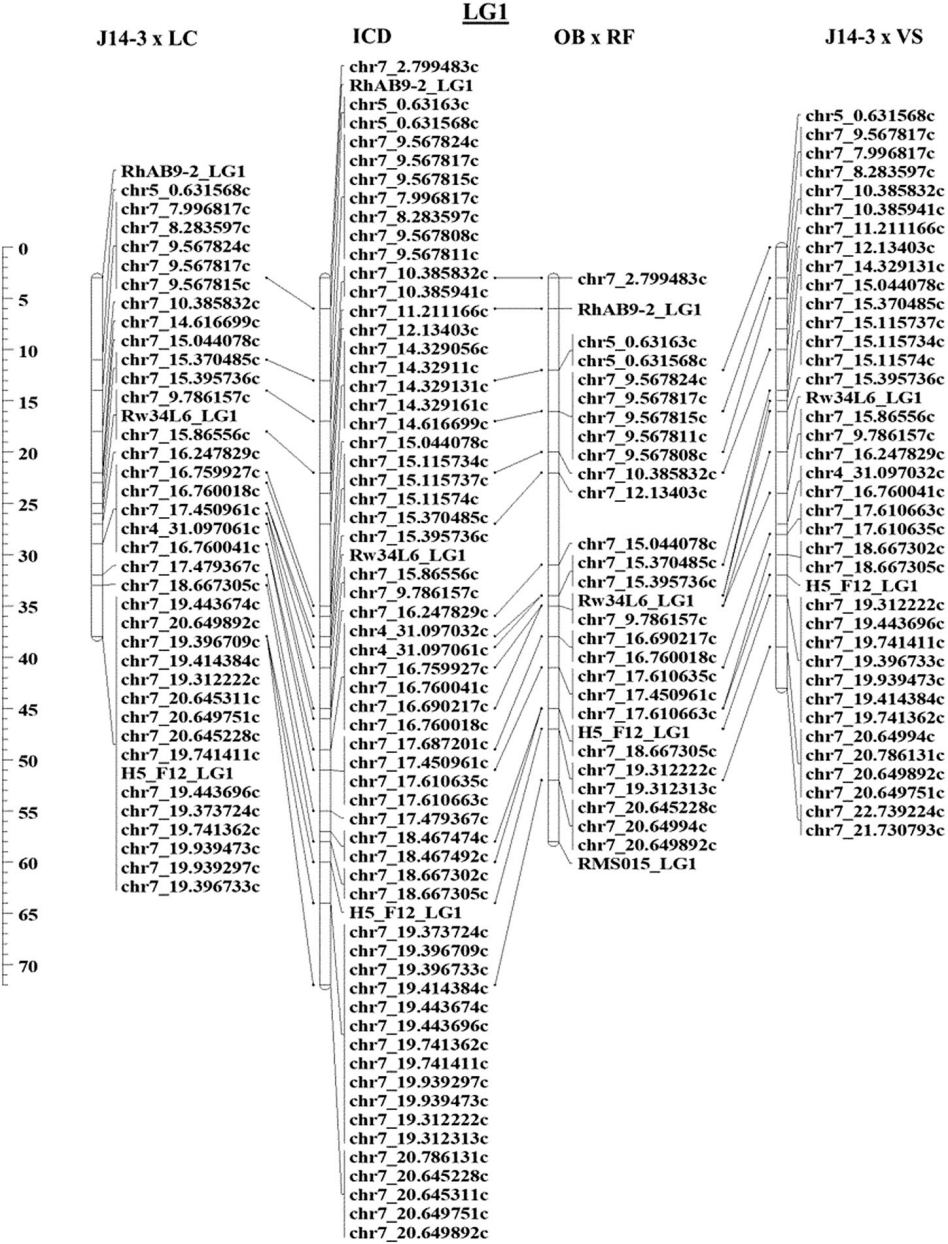
LG1 partial comparison for J14-3×LC, J14-3×VS, OB×RF, and ICD of the diploid rose. The full set of LG1 markers can be found in Supplementary Figure 10.

The ICD was developed based on three bi-parental populations. The comparison of the LGs of all four different maps shows excellent collinearity with only a few rearrangements supporting the use of the GBS protocol for producing high quality markers for genetic map construction (Fig. 2, part of LG1 only; the complete LG1 and all other LGs can be found in Supplementary Figures 10–16; SNP markers and cM positions for the four maps mentioned in this paper can be found in Supplementary Table 3).

### Synteny among diploid rose and *Fragaria vesca*

**Fig. 3:**
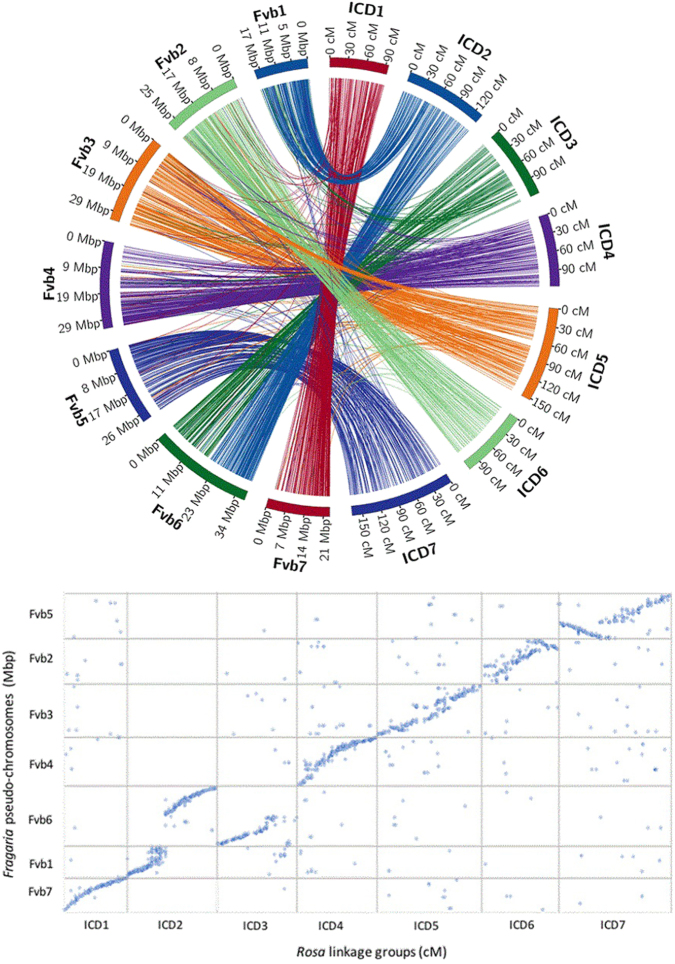
Synteny and collinearity between diploid *Rosa* and *Fragaria*. **a** Circos plot depicting the syntenic relationship between the rose and strawberry linkage groups based on the mapping of rose GBS reads to the *Fragaria vesca* genome sequence. Rose and strawberry were designated as ICD (right) and Fvb (left). Fvb numbering corresponds to the *F. vesca* pseudo-chromosomes with distances in Mb. Rose ICD homologs containing the SNP markers per linkage group are shown with distances in cM. **b** Dot plot comparing rose LGs (ICD) in cM and Fvb assembly physical positions in Mbp. Each dot represents one SNP marker

There was a high level of synteny among the LGs of diploid *Rosa* and strawberry (*Fragaria vesca*) (Fig. 3a). The *F. vesca* genome was used as the “proxy” genome for mapping and SNP detection since a rose reference genome is not presently available. When we grouped and mapped the SNP markers to their respective physical locations on the strawberry assembly, we detected one minor chromosomal inversion close to the telomere on *Rosa* LG6, one major inversion on *Rosa* LG7, and one translocation between diploid *Rosa* LGs 2 and 3 and *Fragaria* LGs 1 and 6 (Fig. 3b). To summarize, *Fragaria* pseudomolecules 7, 4, 3, 2, and 5 correspond to the *Rosa* ICD LGs 1, 4, 5, 6, and 7, respectively. The major translocation seen among LGs 2 and 3 of *Rosa* uncovered that *Rosa* LG2 is composed of *F. vesca* pseudomolecule 1 and a portion of 6, whereas the remainder of *F. vesca* pseudomolecule 6 makes up the majority of *Rosa* LG3. These patterns were consistent across the four maps constructed here (Table 5) and agree with previous studies^14,15^.

**Table 5:** Synteny comparison between diploid *Rosa* and *Fragaria vesca*

LGs	Populations	*Fragaria vesca*						
		1	2	3	4	5	6	7
Diploid *Rosa*								
1	J14-3×LC	2	4	4	4	8	0	**164**
	J14-3×VS	0	3	1	6	5	0	**144**
	OB×RF	0	3	1	1	2	1	**91**
	ICD	2	8	5	9	10	1	**309**
2	J14-3×LC	**136**	0	0	0	0	**134**	0
	J14-3×VS	**160**	0	0	0	0	**136**	0
	OB×RF	**197**	0	0	0	0	**170**	0
	ICD	**380**	0	0	0	0	**371**	0
3	J14-3×LC	14	0	3	4	1	**172**	0
	J14-3×VS	11	1	4	0	0	**104**	1
	OB×RF	8	1	4	2	1	**64**	0
	ICD	27	2	8	5	1	**292**	1
4	J14-3×LC	1	9	9	**173**	2	4	0
	J14-3×VS	4	3	7	**205**	1	3	0
	OB×RF	6	7	7	**198**	0	2	0
	ICD	11	15	19	**465**	3	6	0
5	J14-3×LC	1	6	**235**	16	4	2	9
	J14-3×VS	2	3	**190**	16	2	3	8
	OB×RF	3	5	**258**	11	9	6	10
	ICD	4	10	**484**	26	15	8	15
6	J14-3×LC	1	**210**	4	2	1	0	1
	J14-3×VS	1	**134**	1	1	0	1	1
	OB×RF	0	**215**	4	1	1	1	2
	ICD	1	**451**	8	4	2	2	3
7	J14-3×LC	4	5	1	18	**194**	2	3
	J14-3×VS	3	6	4	21	**222**	1	2
	OB×RF	1	5	8	16	**210**	1	0
	ICD	7	11	12	25	**460**	4	5

The number of markers from each diploid rose linkage group and the consensus map that corresponded to the *Fragaria* v2.0a1 pseudomolecule assembly is shown. Groups of markers strongly indicating the syntenic linkage groups between *Rosa* and *Fragaria* are shown in bold.

## Discussion

### Single map construction

We constructed three individual genetic maps for two half sib (J14-3×LC, J14-3×VS), and one unrelated (OB×RF) highly heterozygous F_1_ populations. The pollen parents of the half sib populations are related as LC is a parent of VS. The breeding line J14-3 (derived from *Rosa wichuriana*) is different from other cultivated parents in various traits, including black spot resistance, growth type, horticultural characteristics, and heat tolerance. The initial breeding focused on combining the everblooming trait from the cultivated germplasm and the high black spot resistance and heat tolerance from *R. wichuriana* into improved breeding selections. These were used in the crosses reported here. These three populations together with other diploid segregating populations sharing common parents or linked via pedigree will help us identify certain QTL and associated markers in our diploid breeding program. With time, some of these selections will be introgressed into the tetraploid rose germplasm within the breeding program.

All the maps contained seven LGs corresponding to the seven base pseudo-chromosomes in rose (*x* = 7). Moreover, consistent collinearity for the seven LGs among the three individual maps and the consensus map was observed (Fig. 2; Supplementary Figures 10–16), and the ordering of anchor SSR markers on our maps was consistent with the *Rosa* ICM map^24^. These results support the high quality and reliability of the maps generated in the present study. Comparing our results to some recent non-SNP-based rose maps^22,24^, marker number and density were increased without length extension using GBS to generate SNP markers and mapping them to the *F. vesca* genome assembly. The overall map length of the three maps are on average ~200 cM shorter than the one of Vukosavljev et al.^14^ and ~70 cM less than the one produced by Bourke et al.^15^

Approximately 7–10% of the initial markers generated from GBS were anchored to the single maps for each cross. Initial grouping of the remaining markers at LOD > 5 in JoinMap® v4.1, produced seven groups representing the seven rose chromosomes in each population. Markers with excessive numbers of double recombination events were eliminated as likely caused by sequencing error. The exclusion of a large proportion of GBS markers is common in other crops as well. For example, only about 10% of the SNPs produced by GBS were kept when constructing the strawberry map^46^, and 4.2% of the starting putative SNPs were retained for grapevine map construction^45^. For small populations, more markers can be incorporated into the consensus map by utilizing more individuals across the populations.

The 26 anchor SSR markers from the rose ICM^24^ used in this study, all initially grouped to their expected LGs supporting the quality of the maps produced herein. However, only twenty of the anchor SSR markers were retained in the final ICD map. The order of most bridge markers was consistent between our maps and the ICM though occasional marker order discrepancies were observed. This could be due to several factors, including segregation distortion, population size, parental genetic background, and scoring errors^22^. Markers displaying segregation distortion (~14%) were present on almost every LG in every population, and clustering of the distorted markers was observed on certain LGs, although it varied among populations. LGs 1, 2, 5, and 6 contained more markers showing segregation distortion than other LGs. This is similar to what has been described in the past, where studies have found 20–22% of the markers on rose maps displaying segregation distortion. This is probably due to the interspecific nature of the crosses but could also be caused by gametophytic incompatibility or genotyping errors^11,22,24^.

We found that the marker density of LG2 was higher and that of LG3 (especially for the J14-3×VS and OB×RF populations) was lower than other LGs. In addition, we observed some large gaps across the LGs. The gaps in the J14-3×VS and OB×RF populations were in the same regions on LG3. Several other large gaps were seen in LG5 for the J14-3×VS population and LGs 3 and 6 for the OB×RF population. This may be the result of not discovering polymorphisms on the strawberry genome, which was used as the “proxy” reference genome or these regions could be dominantly homozygous. Alternatively, we used a methylation sensitive restriction enzyme to digest the rose genomic DNA, and low marker coverage would be expected in repetitive regions containing methylated residues which is seen in other plant species^42^. The present results showed that *Rosa* LG2 was syntenic with *Fragaria* pseudomolecules 1 and 6, and *Rosa* LG3 was syntenic with the remaining portion of *Fragaria* pseudomolecule 6. Fewer markers were mapped onto *Rosa* LG3 and this could be due to the fact that some of the *Rosa* LG3 markers were grouped with those from *Rosa* LG2. However due to the lack of a rose reference genome at present we cannot confirm this information. In apple, because of a genome-wide duplication^55^, the first step in creating the linkage map was to assign groups manually according to the physical position of the markers^56^. As a rose whole-genome sequence becomes available, it will be possible to more accurately assign markers to groups, and determine whether the low number of markers assigned and mapped to *Rosa* LG3 is due to the genetic nature of rose (e.g., repetitive regions) or an incorrect grouping issue.

A few minor marker inversions on LGs were observed among individual maps and the consensus map (Supplementary Figures 10–16). This could be partly explained by the diverse genetic backgrounds in these populations. The inconsistency of some markers can be explained by the tight linkage among different marker pairs, inadequate data (missing data), and differences in segregation information among markers and populations^57^. But overall, no major chromosomal rearrangements were observed across populations.

### Consensus map construction

Over eight hundred bridge markers linked three individual maps into one consensus map containing 820 bin markers (3507 markers including those that co-segregated) covering 892 cM. A comparison between the rose ICD and ICM maps^24^ showed that all the anchor SSRs were mapped to the same linkage groups at similar locations. The total map length of the ICD map was longer and marker number was significantly higher than in previous studies^11,21,24^; we extended the genome coverage (LG length) for LG2 to LG7, whereas the length of LG1 remained the same^24^. The ICD map contains approximately one bin marker every cM, which increased the resolution of the rose genetic map substantially. Regions containing large gaps with no marker coverage in some individual maps were covered in the consensus map, including the lower 15 cM of LG3 and the middle 15 cM of LG5 for the J14-3×VS population and the upper 15 cM and lower 20 cM of LG6 for the OB×RF population. In addition, the marker coverage of LG3 was greatly improved in the ICD map as compared to the individual maps. The extended length of the map may reflect an improved coverage for the rose genome or it is also possible that the genetic distances between markers and the length of LGs were inflated by MergeMap^58^.

Markers with similar segregation patterns were distributed along each LG. The clustering of markers is likely explained by the fact that a large number of markers were mapped on a relatively small number of individuals^24,46^. Few inversions were observed across individual maps and the ICD, and this may be attributed to the small population sizes or the fact that different recombination rates are present among populations^57,59^. Still, some gaps were evident. As the gaps in LG3 and LG5 were located near the middle of the LG, those gaps may be caused by the lack of markers covering heterochromatic pericentromeric regions^60^. This consensus map will serve as one of the basic components required in a pedigree-based QTL analysis (FlexQTL™) to facilitate marker–trait association studies^61^.

### Synteny between *Rosa* and *Fragaria*

Synteny among several Rosaceae crops has been reported in many studies, including *Prunus* crops themselves (almond, peach, apricot, and cherry)^26,28^, *Prunus* and *Malus* (apple)^28^, *Prunus*, *Fragaria* (strawberry) and *Malus*^30^, *Fragaria* and *Prunus*^27^, *Malus* and *Pyrus* (pear)^29^, and *Rosa* (rose) and *Fragaria*^14,15,20^. Our genome-wide comparative analysis with the thousands of SNPs mapped to the diploid *Rosa* LGs and physically located on the *F. vesca* (Fvb) genome further confirmed the high level of synteny among these two genomes. *Rosa* LGs 1, 4, 5, 6, 7 are syntenic to *Fragaria* pseudomolecules 7, 4, 3, 2, and 5, respectively. In addition, a major translocation and fission/fusion occurred between *Rosa* LGs 2 and 3 with *Rosa* LG2 composed of *Fragaria* pseudomolecule 1 (one of the smallest strawberry pseudomolecules) combined with a part of *Fragaria* pseudomolecule 6 (one of the largest strawberry pseudomolecules)^35,46^. The remainder of *Fragaria* pseudomolecule 6 is syntenic to *Rosa* LG3. The syntenic relationship between *Fragaria* and *Rosa* supports a proposed evolutionary relationship among the Rosaceae genomes.

## Conclusion

By mapping sequence-based co-dominant markers (SSR and SNP), we have illustrated the highly conserved synteny between diploid *Rosa* and *Fragaria*, and created a dense SNP-based consensus map for our rose germplasm. This high synteny will facilitate the ability to study the genetics and QTLs between two species and provide a better understanding of the evolution of the Rosaceae. Although we successfully used the *Fragaria* reference genome to find SNPs among *Rosa* sequence data, the accessibility of a rose reference genome that is currently being developed will provide a better view of gene positions and improve the coverage and confidence of the maps created herein. The development of reliable genetic markers for desirable traits in rose will accelerate the introgression of important traits from wild diploid rose species into the genetic background of modern roses and allow the pyramiding of desired traits. The three mapping populations created for this study are segregating for a number of traits including black spot disease response, growth type, plant architecture, and other horticultural traits. Therefore, the genetic maps created in this study will serve as a tool for QTL analysis for many important traits. Those traits segregating in only one population can be mapped using the more traditional bi-parental QTL mapping approach, whereas those traits segregating in multiple populations can be mapped using the ICD map and software such as FlexQTL™^61^. The successful application of GBS on diploid rose may shed light on tetraploid rose as well, but allele dosage is a challenge to address.

### Data availability

Custom perl and python scripts used in the bioinformatics processing of this project can be found in the Dryad Digital Depository, doi:10.5061/dryad.k2do5. Sequence files for all individual rose samples are available at the NCBI Short Read Archive under BioProject PRJNA412522, accessions SAMN07716066-SAMN07716304.

## Electronic supplementary material

Summary of Supplementary Files

Supplementary File 1

Supplementary Table 1

Supplementary Table 2

Supplementary Table 3

Supplementary Figure 1

Supplementary Figure 2

Supplementary Figure 3

Supplementary Figure 4

Supplementary Figure 5

Supplementary Figure 6

Supplementary Figure 7

Supplementary Figure 8

Supplementary Figure 9

Supplementary Figure 10

Supplementary Figure 11

Supplementary Figure 12

Supplementary Figure 13

Supplementary Figure 14

Supplementary Figure 15

Supplementary Figure 16
