# Supplementary material for: Genotyping-by-sequencing application on diploid rose and a resulting high-density SNP-based consensus map
Source: Hortic Res. 2018 Apr 1;5:17. doi: 10.1038/s41438-018-0021-6 (PMC5878828; doi:10.1038/s41438-018-0021-6)
Supplement: Supplementary file 1 — Summary of Supplementary Files [file 41438_2018_21_MOESM1_ESM.docx]

Summary of Supplementary Files

All supplementary files are provided as MS Word or MS Excel documents (.docx or xlsx)

Supplementary File 1 contains information on stock solution preparation and DNA extraction for rose DNA.

Supplementary Table 1 contains information on the SSRs used in map construction including primer sequences, linkage group location and product size in the parents used for this study.

Supplementary Table 2 contains the allele calls for each of the markers in the three diploid rose populations.

Supplementary Table 3 contains the markers used to create each individual population map as well as the ICD map along with the cM position of the marker along each of the 7 rose linkage groups.

Supplementary Figures 1-2 show the 7 linkage maps of the integrated consensus map for diploid rose (ICD).

Supplementary Figures 3-4 show the 7 linkage maps from the J14-3 x LC mapping population.

Supplementary Figures 5-6 show the 7 linkage maps from the J14-3 x VS mapping population.

Supplementary Figures 7-8 show the 7 linkage maps from the OB x RF mapping population.

Supplementary Figure 9 shows a map for the integrated consensus map for diploid rose using bin markers.

Supplementary Figures 10-16 show the collinearity of each linkage group (LG1 through LG7) among the 3 individual population maps and the integrated consensus map.
