# Supplementary material for: Genotyping-by-sequencing application on diploid rose and a resulting high-density SNP-based consensus map
Source: Hortic Res. 2018 Apr 1;5:17. doi: 10.1038/s41438-018-0021-6 (PMC5878828; doi:10.1038/s41438-018-0021-6)
Supplement: Supplementary file 2 — Supplementary File 1 [file 41438_2018_21_MOESM2_ESM.docx]

Supplementary File 1. Stock solution preparation and DNA extraction from rose leaf tissue.

**2X CTAB buffer** (100 ml):
 2% CTAB - 2.00 g
 1.4 M NaCl - 8.12 g
 20 mM EDTA, pH 8.0 - 4 ml of 0.5 M
 100 mM Tris HCl, pH 8.0 - 10 ml of 1.0 M
 1% PVP-40 (polyvinylpyrollidone, M.W. 40,000) - 1.00 g
 β-Mercaptoethanol - 200 μL (add before use)

**Note**: CTAB is difficult to dissolve. Do not add β-Mercaptoethanol until ready to use.

**0.5M EDTA, pH 8** (1000 ml):
 EDTA (Disodium ethylenediaminetetraacetate·2H2O) - 186.1 g

**Preparation**: Add 186.1 g of EDTA to 200 mL of water. Stir vigorously on a magnetic stirrer. Adjust the pH to 8 with NaOH (~20 g of NaOH pellets), then adjust volume of the solution to 1000 mL with water.

**Note**: EDTA will not go into solution until the pH of the solution is adjusted to approximately 8 by the addition of NaOH.

**1.0 M Tris HCl, pH 8** (1000 ml):
 Tris (Hydroxymethyl) Aminomethane - 121.14 g

**Preparation**: Dissolve 121.14 g of Tris in 800 mL of water. Adjust the pH to 8 by adding HCl (~42 mL of concentrated HCl). Allow the solution to cool to room temperature before making final adjustment to the pH. Adjust volume of the solution to 1000 mL with water.

**TE** (100 mL):
 10 mM Tris·HCl - 1.0 mL of 1.0 M
 1 mM EDTA - 0.5 mL of 0.5 M

**Note**: Bring solution to 100 mL with nanopure water.

**CIA** (100 mL):
 Chloroform - 96 mL
 Isoamyl Alcohol - 4 mL

**Note**: Store CIA in dark at room temperature.

Modified Rose DNA Extraction Procedure - High Throughput Method

1. Harvest new growth tissues (usually 10-15 folded leaves, depends on leaf size) up to 1/3 volume of [FastPrep® Lysing Matrix Tubes](http://www.mpbio.com/index.php?cPath=2_77_425) (Lysing Matrix A catalog # 116910050), immediately place tube in liquid nitrogen store in -80°C for later use

2. Prepare 2X CTAB buffer according to ‘stock solution preparation’ document. Add 200 µl β-Mercaptoethanol before use (once added, the buffer can be used for 3-4 days and store at 4°C) Warm at 65°C prior to use.

3. Add 800 µL of pre-warmed 2X CTAB buffer to Fast Prep tubes arrayed in a 96 well micro tube rack. Make note of the sample names in the proper array.

4. Grind the samples in the Geno Grinder with settings at 1500 rpm for 3 minutes 2- 3 times.

5. Place tubes in water bath for 1-1.5 hours

6. Remove samples from water bath and cool on ice 1-2 minutes.

7. Centrifuge at 13000 rpm for 30 minutes and collect supernatant into new 1.2 ml library tubes in rack – very important to double check the array prior to this transfer step.

8. Add 400 µl of CIA to each tube (working under hood) tightly cap the tubes with strip caps, place in clamp apparatus then invert to mix

9. Centrifuge racks in table top centrifuge at 3500 rpm for 15 minutes

10. Collect top layer and place in a new 1.2 ml library tubes in rack – very important to double check the array prior to this transfer step.

11. Add 600 µl of CIA to each tube (working under hood) tightly cap the tubes with strip caps, place in clamp apparatus then invert to mix

12. Centrifuge racks in table top centrifuge at 3500 rpm for 15 minutes

13. Collect top layer and place in a new 1.2 ml library tubes in rack – very important to double check the array prior to this transfer step.

14. Add 600 µl of cold isopropanol (-20°C) to each 1.2 ml library tube

15. Mix well by pipetting. DNA may precipitate during this step

16. Place samples in freezer (-20°C) overnight or (-80°C) for 3 hours

17. Centrifuge racks in table top centrifuge at 3500 rpm for 45-60 minutes

18. Carefully remove supernatant with pipet.

19. Wash pellet with 300 µl 70% EtOH (need to wash thoroughly by breaking down the pellet). After washing, centrifuge at 4000 for 5 minutes - repeat until you see the pellet become colorless.

20. Remove EtOH and allow to air dry at room temperature

21. Add 90 µl of TE to each tube, pipet to dissolve the DNA.

22. RNase treat by adding 1 µl Ambion RNase (AM2288), incubate for 1 hour at 37°C

23. Follow Zymo OneStep™ PCR Inhibitor Removal Kit (Catalog #D6035 (96-well)) protocol to clean DNA

24. Store samples at -20°C

Final concentration should be around 100-200 ng/µl
