# Supplementary material for: Genotyping-by-sequencing application on diploid rose and a resulting high-density SNP-based consensus map
Source: Hortic Res. 2018 Apr 1;5:17. doi: 10.1038/s41438-018-0021-6 (PMC5878828; doi:10.1038/s41438-018-0021-6)
Supplement: Supplementary file 3 — Supplementary Table 1 [file 41438_2018_21_MOESM3_ESM.docx]

Supplementary Table 1. PCR product sizes (bp) of 26 labeled SSR primers for the parents of the J14-3 x VS, J14-3 x LC and OB x RF diploid rose populations.

| **SSR** | **Primer (5'-3')** | **Linkage group** | **J14-3** | **LC** | **OB** | **RF** | **VS** |
| --- | --- | --- | --- | --- | --- | --- | --- |
| **BFACT47_LG3** | F: ATCGTACCTATGCATCATCTGC | LG3 | 148, 159 | - | 144, 148 | 143, 147 | 147, 165 |
|  | R: AGGCAGGACCATCAACTAAGAG |  |  |  |  |  |  |
| **CL2845_LG5** | F: ACAACCCGTAAAACGACCTG | LG5 | 295, 304 | 295, 306 | 295 | - | 295, 306 |
|  | R: ATATGGTGCCTTTGGTGGAA |  |  |  |  |  |  |
| **CL2980_LG6** | F: CCCTATTCGATTTCGAGTGC | LG6 | 122, 225, 228 | 122 | 127, 222 | 122, 225, 231 | 125, 223, 235 |
|  | R: ACTTGGCTCGACGGATACAC |  |  |  |  |  |  |
| **CL2996_LG2** | F: GCCACCATAGCCAGAGACAT | LG2 | - | 181, 187 | 178, 187 | 178, 184 | 175, 187 |
|  | R: GGGCAGAGAAGAAGTTGACG |  |  |  |  |  |  |
| **CL3881_LG4** | F: GACAACGACCACACCACTTG | LG4 | 237, 245 | 238, 240 | 237, 240 | 245 | 232, 249 |
|  | R: CCAAAGCAACATTGTCAAAAGA |  |  |  |  |  |  |
| **CTG21_LG3** | F: CACAGTTTCCATTAACACAGCA | LG3 | 121, 131 | - | 121, 133 | 120, 123 | 124, 133 |
|  | R: CAAGAGGAGGCAAGAGGATG |  |  |  |  |  |  |
| **H5_F12_LG1** | F: CACAGAAACGAAGCGCAGTA | LG1 | 132 | 132, 141 | 132, 138 | 132, 138 | 132, 141 |
|  | R: GCTCGAAGAAGTCCTGGATG |  |  |  |  |  |  |
| **Rh48_LG2** | F: GATAGTTTCTCTGTACCCCACCTA | LG2 | 105, 118 | 96, 106, 119 | 96, 118 | 97, 117, 144 | 96, 118, 135 |
|  | R: TTGACCAGCTGCAACAAAATTAGA |  |  |  |  |  |  |
| **Rh50_LG3** | F: TGATGAAATCATCCGAGTGTCAG | LG3 | 96, 310, 337 | 304 | 304, 333 | 304, 310 | 304, 310 |
|  | R: TCACTTTCATTGGAATGCCAGAAT |  |  |  |  |  |  |
| **Rh58_LG3** | F: ACAATTTAGTGCGGATAGAACAAC | LG3 | 240, 264 | 289 | 248, 289 | 252 | 248, 289 |
|  | R: GGAAAGCCCGAAAGCGTAAGC |  |  |  |  |  |  |
| **Rh72_LG7** | F: CCAAAAGACGCAACCCTACCATAA | LG7 | 263, 269 | 261, 263 | 252, 261 | 261, 269 | 261, 277 |
|  | R: TCAAAACGCATGATGCTTCCACTG |  |  |  |  |  |  |
| **Rh93_LG5** | F: GCTTTGCTGCATGGTTAGGTTG | LG5 | 129, 190, 243 | 130, 190 | 130, 190, 237 | 130, 190, 243 | 129, 189, 267 |
|  | R: TTCTTTTTGTCGTTCTGGGATGTG |  |  |  |  |  |  |
| **RhAB9-2_LG1** | F: GTCAATTTGTGCATAAGCTC | LG1 | 95, 97 | 94, 99 | 94, 113 | 95, 101 | 113 |
|  | R: GTGAGAACAGATGAGAAATG |  |  |  |  |  |  |
| **RhABT12_LG4** | F: CAAGTTTGTCTCCTTGGACC | LG4 | 162, 198 | 151, 175 | 151, 165 | 151, 202 | 151, 156 |
|  | R: CATAGATGATTATCCTAGAGCC |  |  |  |  |  |  |

| **SSR** | **Primer (5'-3')** | **Linkage group** | **J14-3** | **LC** | **OB** | **RF** | **VS** |
| --- | --- | --- | --- | --- | --- | --- | --- |
| **RMS001_LG7** | F: TTCAAAATTGCTGCCCCCTTAG | LG7 | 229, 241 | 226, 229, 231, 233, 234 | 222, 226, 231, 234 | - | 229, 232 |
|  | R: TACCAGTTGAGTGAGAAATAGTT |  |  |  |  |  |  |
| **RMS003_LG7** | F: TGGGAAAGGGAAAGCAACA | LG7 | 160, 163 | 156, 170 | 150, 170 | 160, 174 | 164, 202 |
|  | R: AAGGTAGGCAGAAGTGACAGACAT |  |  |  |  |  |  |
| **RMS015_LG1** | F: TAATGTAGGCAGATATAAAGGAGT | LG1 | 131, 166 | 132, 154, 172 | 132, 154, 172 | 130, 162 | 131, 164, 182 |
|  | R: GCAGCTGCACAACAAGGAA |  |  |  |  |  |  |
| **RMS043_LG7** | F: GATCAAAGATGGGTTCTCCTCTC | LG7 | 130, 205, 220 | 130, 205 | 129, 205, 214 | 129 | 217 |
|  | R: AGGGGAATCTTTGAAAGTCGTTC |  |  |  |  |  |  |
| **Rw11E5_LG6** | F: GATACCGCGAAGGTGTAGT | LG6 | 152, 164, 172, 182 | 167 | 154,167, 172, 179 | 152, 167, 170 | 139, 168 |
|  | R: GAGTGAAAACTCTGCAATCA |  |  |  |  |  |  |
| **Rw12J12_LG3** | F: CAGTGTCCATGCTGACGAGT | LG3 | 158, 171 | 170 | 145, 170 | 164, 171 | 160, 164 |
|  | R: TGCTCCTGTTTTCTCTTTGCT |  |  |  |  |  |  |
| **Rw14H21_LG5** | F: ATCATGTGCAGTCTCCTGGT | LG5 | 118, 145 | 118, 120 | 118, 123 | 118, 153 | 118, 120 |
|  | R: AATTGTGGGCTGGAAATATG |  |  |  |  |  |  |
| **Rw22B6_LG7** | F: ACAGTGAGTTGTTCGCTTCT | LG7 | 133, 139 | 134 | 131, 133 | 134 | 133 |
|  | R: TTCATTGCTAGGAAGCAGTA |  |  |  |  |  |  |
| **Rw34L6_LG1** | F: CTCCTTTAGACTCGGGACCA | LG1 | 132, 212 | 130, 209 | 131, 210? | 127, 206 | 129, 208 |
|  | R: CAGGCACGCCATTTCTAACT |  |  |  |  |  |  |
| **Rw35C24_LG3** | F: GGCGAATCGAGATTCAGAGA | LG3 | 254, 258 | 246, 261 | 246, 258 | 248, 250 | 246, 252 |
|  | R: GGATTAGCCCAAGTCCAGGT |  |  |  |  |  |  |
| **Rw55E12_LG4** | F: CGGTGGTTGGACATTAAAGC | LG4 | 133, 180 | 130, 170, 182 | 130, 165, 182 | 130, 170, 182 | 132, 178 |
|  | R: GGAGGCAACAGCACACTCTC |  |  |  |  |  |  |
| **Rw5G14_LG7** | F: TGGTTTGGGGTTTTGTGTCT | LG7 | 234, 250 | 242, 250 | 250, 252 | 227, 238 | 227, 252 |
|  | R: GCACAGTCTCCACCTGACAA |  |  |  |  |  |  |
