# Supplementary material for: Genotyping-by-sequencing application on diploid rose and a resulting high-density SNP-based consensus map
Source: Hortic Res. 2018 Apr 1;5:17. doi: 10.1038/s41438-018-0021-6 (PMC5878828; doi:10.1038/s41438-018-0021-6)
Supplement: Supplementary file 6 — Supplementary Figure 1 [file 41438_2018_21_MOESM6_ESM.docx]

Supplementary Figure 1. LG1-4 of the integrated consensus map for diploid roses (ICD). Anchor SSR markers are shown in red and underlined.
