# Supplementary material for: Genotyping-by-sequencing application on diploid rose and a resulting high-density SNP-based consensus map
Source: Hortic Res. 2018 Apr 1;5:17. doi: 10.1038/s41438-018-0021-6 (PMC5878828; doi:10.1038/s41438-018-0021-6)
Supplement: Supplementary file 7 — Supplementary Figure 2 [file 41438_2018_21_MOESM7_ESM.docx]

Supplementary Figure 2. LG5-7 of the integrated consensus map for diploid roses (ICD). Anchor SSR markers are shown in red and underlined.
