# Supplementary material for: Genotyping-by-sequencing application on diploid rose and a resulting high-density SNP-based consensus map
Source: Hortic Res. 2018 Apr 1;5:17. doi: 10.1038/s41438-018-0021-6 (PMC5878828; doi:10.1038/s41438-018-0021-6)

Supplementary Figure 8. LG5-7 of OB x RF. Anchor SSR markers are shown in red and underlined. Marker distortion is indicated beside marker names: *, **, ***, ****, *****, ****** represent significance at *p*≤ 0.1, 0.05, 0.01, 0.001, 0.005, 0.001, or 0.0005, respectively, based on a Chi-square test.


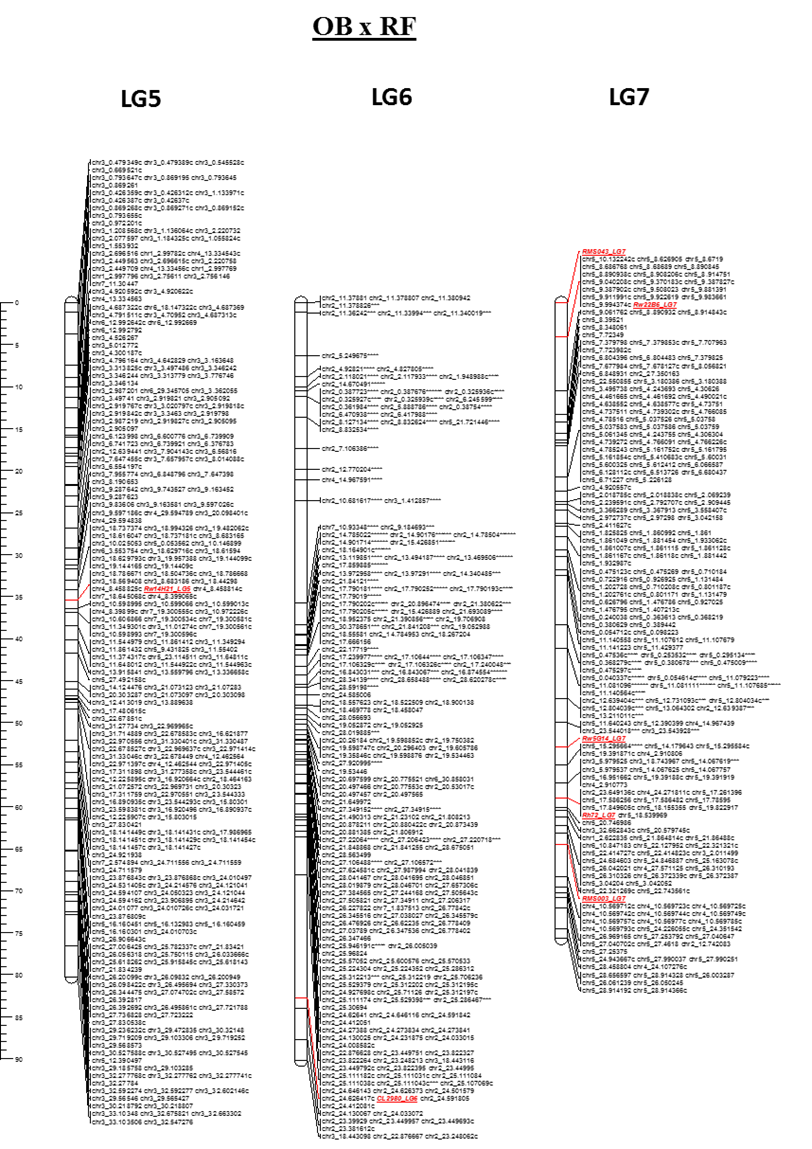

Supplement: Supplementary file 13 — Supplementary Figure 8 [file 41438_2018_21_MOESM13_ESM.docx]
