# Supplementary figures and images for: Genotyping-by-sequencing application on diploid rose and a resulting high-density SNP-based consensus map
Source: Hortic Res. 2018 Apr 1;5:17. doi: 10.1038/s41438-018-0021-6 (PMC5878828; doi:10.1038/s41438-018-0021-6)

Supplementary Figure 9. Integrated consensus map for diploid rose (ICD) using bin markers.


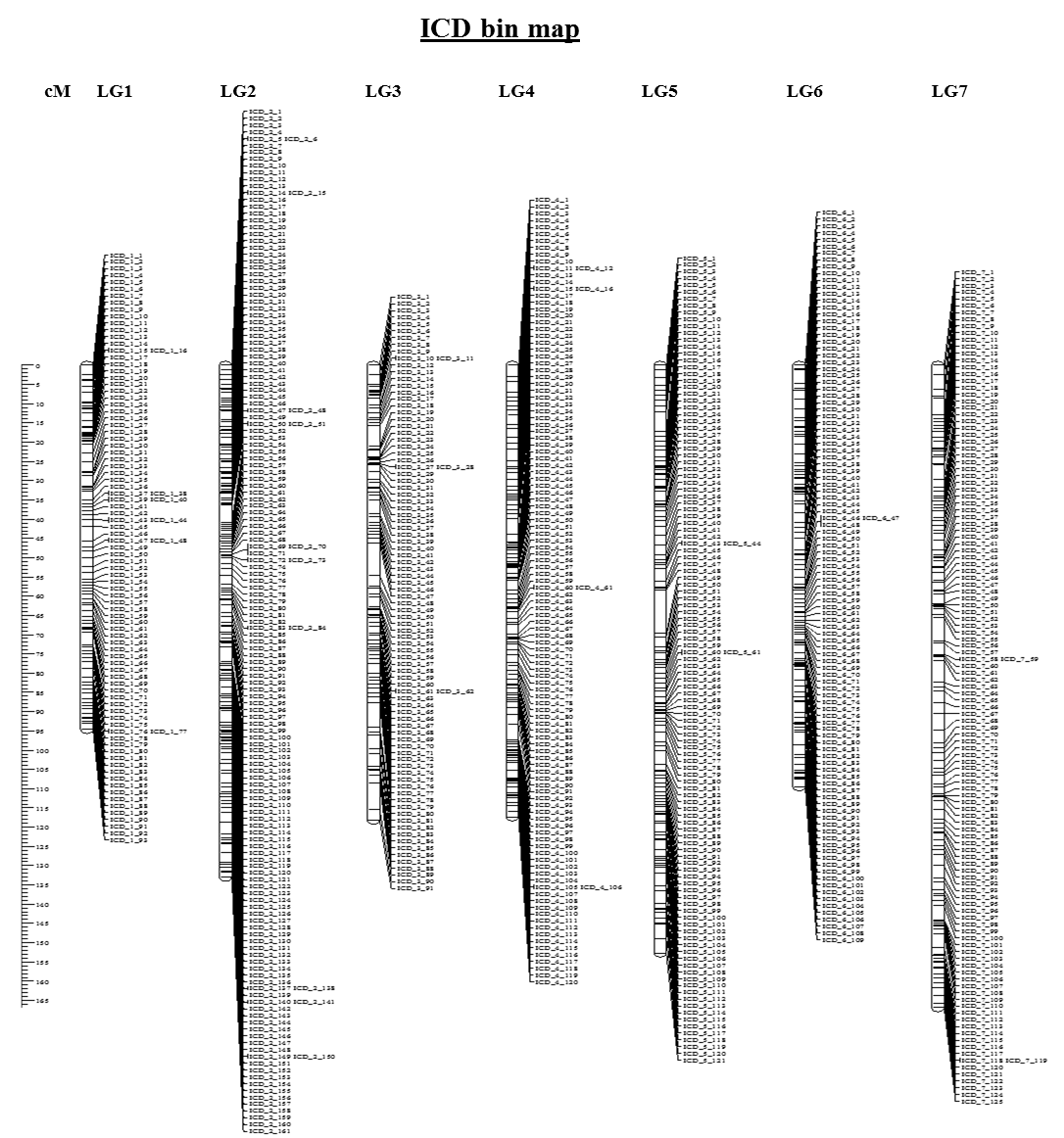

Supplement: Supplementary file 14 — Supplementary Figure 9 [file 41438_2018_21_MOESM14_ESM.docx]
