# Supplementary material for: Genotyping-by-sequencing application on diploid rose and a resulting high-density SNP-based consensus map
Source: Hortic Res. 2018 Apr 1;5:17. doi: 10.1038/s41438-018-0021-6 (PMC5878828; doi:10.1038/s41438-018-0021-6)
Supplement: Supplementary file 15 — Supplementary Figure 10 [file 41438_2018_21_MOESM15_ESM.docx]

Supplementary Figure 10. Collinearity of LG1 among the three individual maps and the consensus map. Anchor SSR markers are shown in red and underlined. Common markers across the maps are linked via black solid lines.
