# Supplementary material for: Genotyping-by-sequencing application on diploid rose and a resulting high-density SNP-based consensus map
Source: Hortic Res. 2018 Apr 1;5:17. doi: 10.1038/s41438-018-0021-6 (PMC5878828; doi:10.1038/s41438-018-0021-6)
Supplement: Supplementary file 17 — Supplementary Figure 12 [file 41438_2018_21_MOESM17_ESM.docx]

Supplementary Figure 12. Collinearity of LG3 among the three individual maps and the consensus map. Anchor SSR markers are shown in red and underlined. Common markers across the maps are linked via black solid lines.
