# Supplementary material for: Genotyping-by-sequencing application on diploid rose and a resulting high-density SNP-based consensus map
Source: Hortic Res. 2018 Apr 1;5:17. doi: 10.1038/s41438-018-0021-6 (PMC5878828; doi:10.1038/s41438-018-0021-6)
Supplement: Supplementary file 21 — Supplementary Figure 16 [file 41438_2018_21_MOESM21_ESM.docx]

Supplementary Figure 16. Collinearity of LG7 among the three individual maps and the consensus map. Anchor SSR markers are shown in red and underlined. Common markers across the maps are linked via black solid lines.
